# Pybel: a Python wrapper for the OpenBabel cheminformatics toolkit

**DOI:** 10.1186/1752-153X-2-5

**Published:** 2008-03-09

**Authors:** Noel M O'Boyle, Chris Morley, Geoffrey R Hutchison

**Affiliations:** 1 Unilever Centre for Molecular Science Informatics, Department of Chemistry, University of Cambridge, Lensfield Road, Cambridge CB2 1EW, UK; 2 Cambridge Crystallographic Data Centre, 12 Union Road, Cambridge CB2 1EZ, UK; 3 OpenBabel Development Team; 4 Department of Chemistry, University of Pittsburgh, Chevron Science Center, 219 Parkman Avenue, Pittsburgh, PA 15260, USA

## Abstract

**Background:**

Scripting languages such as Python are ideally suited to common programming tasks in cheminformatics such as data analysis and parsing information from files. However, for reasons of efficiency, cheminformatics toolkits such as the OpenBabel toolkit are often implemented in compiled languages such as C++. We describe Pybel, a Python module that provides access to the OpenBabel toolkit.

**Results:**

Pybel wraps the direct toolkit bindings to simplify common tasks such as reading and writing molecular files and calculating fingerprints. Extensive use is made of Python iterators to simplify loops such as that over all the molecules in a file. A Pybel Molecule can be easily interconverted to an OpenBabel OBMol to access those methods or attributes not wrapped by Pybel.

**Conclusion:**

Pybel allows cheminformaticians to rapidly develop Python scripts that manipulate chemical information. It is open source, available cross-platform, and offers the power of the OpenBabel toolkit to Python programmers.

## Background

Cheminformaticians often need to write once-off scripts to create extract data from text files, prepare data for analysis or carry out simple statistics. Scripting languages such as Perl, Python and Ruby are ideally suited to these day-to-day tasks [1]. Such languages are, however, an order of magnitude or more slower than compiled languages such as C++. Since cheminformaticians regularly deal with molecular files containing thousands of molecules and many cheminformatics algorithms are computationally expensive, cheminformatics toolkits are typically written in compiled languages for performance.

OpenBabel is a C++ toolkit with extensive capabilities for reading and writing molecular file formats (over 80 are supported) as well as for manipulating molecular data [2]. Many standard chemistry algorithms are included, for example, determination of the smallest set of smallest rings, bond order perception, addition of hydrogens, and assignment of Gasteiger charges. In relation to cheminformatics, OpenBabel supports SMARTS searching [3], molecular fingerprints [4] (both Daylight-type, and structural-key based), and includes group contribution descriptors for LogP [5], polar surface area (PSA) [6] and molar refractivity (MR) [5].

Of the current popular scripting languages, Python [7] is the *de-facto *standard language for scripting in cheminformatics. Several commercial cheminformatics toolkits have interfaces in Python: OpenEye's closed-source successor to OpenBabel, OEChem [8], is a C++ toolkit with interfaces in Python and Java; Rational Discovery's RDKit [9], which is now open source, is a C++ cheminformatics toolkit with a Python interface; the Daylight toolkit [10] from Daylight Chemical Information Systems, written in C, only has Java and C++ wrappers but PyDaylight [11], available separately from Dalke Scientific, provides a Python interface to the toolkit; the Cambios Molecular Toolkit [12] from Cambios Consulting is a commercial C++ toolkit with a Python interface. There are also toolkits entirely implemented in Python: Frowns [13], an open source cheminformatics toolkit by Brian Kelley, and PyBabel [14], an open source toolkit included in the MGLTools package from the Molecular Graphics Labs at the Scripps Research Institute. Note that the latter is not related to the OpenBabel project; rather its name derives from the fact that its aim was to implement in Python some of the functionality of Babel v1.6 [15], a command-line application for converting file formats which is a predecessor of OpenBabel.

Here we describe the implementation and application of Pybel, a Python module that provides access to the OpenBabel C++ library from the Python programming language. Pybel builds on the basic Python bindings to make it easier to carry out frequent tasks in cheminformatics. It also aims to be as 'Pythonic' as possible; that is, to adhere to Python language conventions and idioms, and where possible to make use of Python language features such as iterators. The result is a module that takes advantage of Python's expressive syntax to allow cheminformaticians to carry out tasks such as SMARTS matching, data field manipulation and calculation of molecular fingerprints in just a few lines of code.

## Implementation

### SWIG bindings

Python bindings to the OpenBabel toolkit were created using SWIG [16]. SWIG (Simplified Wrapper and Interface Generator) is a tool that automates the generation of bindings to libraries written in C or C++. One of the advantages of SWIG compared to other automated wrapping methods such as Boost.Python [17] or SIP [18] is that SWIG also supports the generation of bindings to several other languages. For example, OpenBabel also uses SWIG to generate bindings for Perl, Ruby and Java. An additional advantage is that SWIG will directly parse C or C++ header files while Boost.Python and SIP require each C++ class to be exposed manually. The input to SWIG is an interface file containing a list of OpenBabel header files for which to generate bindings. Using the signatures in the header files, SWIG generates a C file which, when compiled and linked with the Python development libraries and OpenBabel, creates a Python extension module, *openbabel*. This can then be imported into a Python script like any other Python module using the "*import openbabel*" statement.

For a small number of C++ objects and functions, it was necessary to add some convenience functions to facilitate access from Python. Certain types of molecule files have additional data present in addition to the connection table. OpenBabel stores these data in subclasses of OBGenericData such as OBPairData (for the data fields in molecule files such as MOL files and SDF files) and OBUnitCell (for the data fields in CIF files). To access the data it is necessary to 'downcast' an instance of OBGenericData to the specific subclass. For this reason, two convenience functions were added to the interface file, one to cast OBGenericData to OBPairData, and one to cast to OBUnitCell. Another convenience function was added to convert a Python list to a C array of doubles, as this type of input is required for a small number of OpenBabel functions.

Iterators are an important feature of the OpenBabel C++ library. For example, OBAtomAtomIter allows the user to easily iterate over the atoms attached to a particular atom, and OBResidueIter is an iterator over the residues in a molecule. The OpenBabel iterators use the dereference operator to access the data, the increment operator to iterate to the next element, and the boolean operator to test whether any elements remain. Iterators are also a core feature of the Python language. However, the iterators used by OpenBabel are not automatically converted into Python iterators. To deal with this, Python iterator classes that wrap the dereference, increment and boolean operators behind the scenes were added to the SWIG interface file, so that Python statements such as "*for attached_obatom in OBAtomAtomIter(obatom)*" work without problem.

### Pybel module

**Figure 1:**
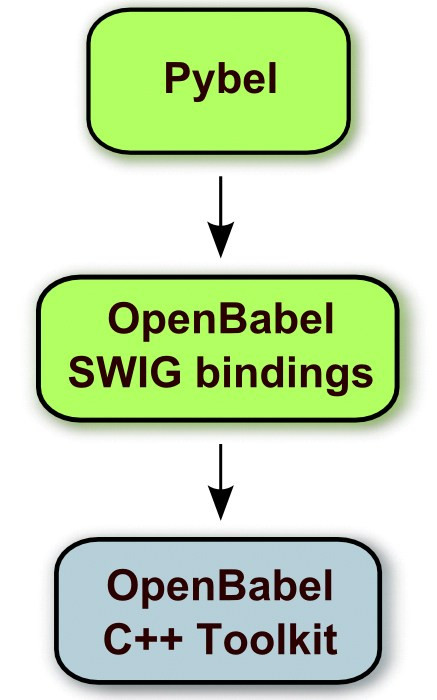
**The relationship between Python modules described in the text and the OpenBabel C++ library**. Python modules are shown in green; the C++ library is shown in blue.

The SWIG bindings provide direct access from Python to the C++ objects and functions in the OpenBabel API (application programming interface). The purpose of the Pybel module is to wrap these bindings to present a more Pythonic interface to OpenBabel (Figure 1). This extra level of abstraction is useful as Python programmers expect Python libraries to behave in certain ways that a C++ library does not. For example, in Python, attributes of an object are often directly accessed whereas in C++ it is typical to call Get/Set functions to access them. A C++ function returning a particular object might require a pointer to an empty object as a parameter, whereas the Python equivalent would not. Even something as simple as differences in the conventions for the case of letters used in variable and method names is a problem, as it makes it more likely for Python programmers to introduce bugs in their code.

One of the key aims of Pybel was to reduce the amount of code necessary to carry out common tasks. This is especially important for a scripting language where programming is often done interactively at a command prompt. In addition, as for any programming language, repeated entry of code for routine and common tasks (so-called 'boilerplate code') is a common cause of errors in code. Reading and writing molecule files is one of the most common tasks for users of OpenBabel but requires several lines of code if using the SWIG bindings. The following code shows how to store each molecule in a multimolecule SDF file in a list called *allmols*:

import openbabel

allmols = []

obconversion = openbabel.OBConversion()

obconversion.SetInFormat("sdf")

obmol = openbabel.OBMol()

notatend = obconversion.ReadFile(obmol, "inputfile.sdf")

while notatend:

   allmols.append(obmol)

   obmol = openbabel.OBMol()

   notatend = obconversion.Read(obmol)

To replace this somewhat verbose code, Pybel provides a *readfile *method that takes a file format and filename and returns molecules using the 'yield' keyword. This changes the method into a 'generator', a Python language feature where a method behaves like an iterator. Iterators are a major feature of the Python language which are used for looping over collections of objects. In Pybel, we have used iterators where possible to simplify access to the toolkit. As a result, the equivalent to the preceding code is:

import pybel

allmols = [mol for mol in pybel.readfile("sdf", "inputfile.sdf")]

The benefits of iterator syntax are clear when dealing with multimolecule files. For single molecule files, however, the user needs to remember to explicitly request the iterator to return the first and only molecule using the *next *method:

mol = pybel.readfile("mol", "inputfile.mol").next()

Pybel provides replacements for two of the main classes in the OpenBabel library, OBMol and OBAtom. The following discussion describes the Pybel Molecule class which wraps an instance of OBMol, but the same design principles apply to the Pybel Atom class. Table 1 summarises the attributes and methods of the Molecule object. By wrapping the base class, Pybel can enhance the Molecule object by providing (1) direct access to attributes rather than through the use of Get methods, (2) additional attributes of the object, and (3) additional methods that act on the object.

**Table 1:** Attributes and methods supported by the Pybel Molecule object

**Attribute**	Description*
OBMol	The underlying OBMol object
atoms	A list of Pybel Atoms
charge	The total charge (GetTotalCharge)
data	A MoleculeData object for access to data fields
dim	The dimensionality of the coordinates (GetDimension)
energy	The heat of formation (GetEnergy)
exactmass	The mass calculated using isotopic abundance (GetExactMass)
flags	The set of flags used internally by OpenBabel (GetFlags)
formula	The stoichiometric formula (GetFormula)
mod	The number of nested BeginModify() calls (Internal use) (GetMod)
molwt	The standard molar mass (GetMolWt)
spin	The total spin multiplicity (GetTotalSpinMultiplicity)
sssr	The smallest set of smallest rings (GetSSSR)
title	The title of the molecule (often the filename) (GetTitle)
unitcell	Unit cell data (if present)

**Method**	
write	Write the molecule to a file or return it as a string
calcfp	Return a molecular fingerprint as a Fingerprint object
calcdesc	Return the values of the group contribution descriptors
__iter__	Enable iteration over the Atoms in the Molecule

*Where a Molecule attribute is a direct replacement for a 'Get' method of the underlying OBMol, the name of the method is given in parentheses.

(1) As mentioned earlier, it is typical in Python to access attribute values directly rather than using Get/Set methods. With this in mind, the Molecule class adds attributes such as *energy*, *formula *and *molwt *(among others) which give the values returned by calling *GetEnergy()*, *GetFormula() *and *GetMolWt()*, respectively on the underlying OBMol (see Table 1 for the full list).

(2) One of the aims of Pybel is to simplify access to some of the most common attributes. With this in mind, an *atoms *attribute has been added which returns a list of the atoms of the molecule as Pybel Atoms. Access to the data fields associated with a molecule has been simplified by creation of a MoleculeData object which is returned when the *data *attribute of a Molecule is accessed. MoleculeData presents a dictionary interface to the data fields of the molecule. Accessing and updating these field is more convoluted if using the SWIG bindings. Compare the following statements for accessing the "comment" field of the variable *mol*, an OBMol:

# Using the SWIG bindings

value = openbabel.toPairData(mol.GetData ["comment"]).GetValue()

# Using Pybel

value = pybel.Molecule(mol).data ["comment"]

It should be noted that all of these attributes are calculated on-the-fly rather than stored for future access as the underlying OBMol may have been modified.

(3) Four additional methods have been added to the Pybel Molecule (Table 1). The first is a *write *method which writes a representation of the Molecule to a file and takes care of error handling. As with reading molecules from files (see above), this method simplifies the procedure significantly compared to using the SWIG bindings directly. In addition, a *calcfp *method and a *calcdesc *method have been added which calculate a binary fingerprint for the molecule, and some descriptor values, respectively. In the OpenBabel library these are not methods of the OBMol, but rather are loaded as plugins (by OBFingerprint.FindFingerprint and OBDescriptor.FindType, respectively) to which an OBMol is passed as input. The *__iter__ *method is a special Python method that enables iteration over an object; in the case of a Molecule, the defined iterator loops over the Atoms of the Molecule. This feature enables constructions such as "*for atom in mol*" where *mol *is a Pybel Molecule.

SMARTS is a query language developed by Daylight Chemical Information Systems for molecular substructure searching [3]. As implemented in the OpenBabel toolkit, finding matches of a particular substructure in a particular molecule is a four step process that involves creating an instance of OBSmartsPattern, initialising it with a SMARTS pattern, searching for a match, and finally retrieving the result:

obsmarts = openbabel.OBSmartsPattern()

obsmarts.Init("[#6] [#6]")

obsmarts.Match(obmol)

results = obsmarts.GetUMapList()

Since a SMARTS query can be thought of as a regular expression for molecules, in Pybel we decided to wrap the SMARTS functionality in an analogous way to Python's regular expression module, *re*. With these changes, the same process takes only two steps, an initialisation step and a search step:

smarts = pybel.Smarts("[#6] [#6]")

results = smarts.findall(pybelmol)

Pybel was not written to replace the SWIG bindings but rather to make it simpler to perform common tasks. As a result, Pybel does not attempt to wrap every single method and class in the OpenBabel library. Because of this, a user may often want to interconvert between an OBMol and a Molecule, or an OBAtom and an Atom. This is quite a straightforward process. A Pybel Molecule can be created by passing an OBMol to the Molecule constructor. In the following example an OBMol is created using the SWIG bindings and then written to a file using Pybel:

obmol = openbabel.OBMol()

a = obmol.NewAtom()

a.SetAtomicNum(6)

a.SetVector(0.0, 1.0, 2.0) # Set coordinates

b = obmol.NewAtom()

obmol.AddBond(1, 2, 1) # Single bond from Atom 1 to Atom 2

pybel.Molecule(obmol).write("mol", "outputfile.mol")

The OBMol wrapped by a Pybel Molecule can be accessed through the *OBMol *attribute. This makes it easy to call a method not wrapped by Pybel, such as *OBMol.NumRotors*, which returns the number of rotatable bonds in a molecule:

mol = pybel.readfile("mol", "inputfile.mol").next()

numrotors = mol.OBMol.NumRotors()

### Documentation and Testing

To minimise programming errors, programs written dynamically-typed languages such as Python should be tested comprehensively. Pybel has 100% code coverage in terms of unit tests, as measured by Ned Batchelder's coverage.py [19]. It also has several doctests, short snippets of Python code included in documentation strings which serve as both examples of usage and as unit tests.

The Pybel API is fully documented with docstrings. These can be accessed in the usual way with the help() command at the interactive Python prompt after importing Pybel: for example, "*help(pybel.Molecule)*". In addition, the OpenBabel Python web page [20] contains a complete description of how to use the SWIG bindings and the Pybel API. The webpage also contains links to HTML versions of the OpenBabel API documentation and Pybel API documentation. The latter is included in Additional File 1.

## Results and Discussion

The principle aim of Pybel is to make it simpler to use the OpenBabel toolkit to carry out common tasks in cheminformatics. These common tasks include reading and writing molecule files, accessing data fields of a molecule, computing and comparing molecular fingerprints and SMARTS matching. Here we present some examples that illustrate how Pybel may be used to carry out common cheminformatics tasks.

### Removal of duplicate molecules

When merging different datasets or as a final step in preprocessing, it may be necessary to identify and remove duplicate molecules. In the following example, only the unique molecules in the multimolecule SDF file "inputfile.sdf" will be written to "uniquemols.sdf". Here we will assume that a unique InChI string (IUPAC International Chemical Identifier) indicates a unique molecule. A similar procedure could be performed using the OpenBabel canonical SMILES format, by replacing "inchi" with "can" in the following:

import pybel

inchis = []

output = pybel.Outputfile("sdf", "uniquemols.sdf")

for mol in pybel.readfile("sdf", "inputfile.sdf"):

   inchi = mol.write("inchi")

   if inchi not in inchis:

      output.write(mol)

      inchis.append(inchi)

output.close()

### Selection of similar molecules

Another common task in cheminformatics is the selection of a set of molecules of similar structure to a target molecule. Here we will assume that structural similarity is indicated by a Tanimoto coefficient [21] of at least 0.7 with respect to Daylight-type (that is, based on hashed paths through the molecular graph) fingerprints. Note that Pybel redefines the | operator (bitwise OR) for Fingerprint objects as the Tanimoto coefficient:

import pybel

targetmol = pybel.readfile("sdf", "targetmol.sdf").next()

targetfp = targetmol.calcfp()

output = pybel.Outputfile("sdf", "similarmols.sdf")

for mol in pybel.readfile("sdf", "inputfile.sdf"):

   fp = mol.calcfp()

   if fp | targetfp >= 0.7:

      output.write(mol)

output.close()

### Applying a Rule of Fives filter

In an influential paper, Lipinski et al. [22] performed an analysis of drug compounds that reached Phase II clinical trials and found that they tended to occupy a certain range of values for molecular weight, LogP, and number of hydrogen bond donors and acceptors. Based on this, they proposed a rule with four criteria to identify molecules that might have poor absorption or permeation properties. This is the Lipinski Rule of Fives, so-called as the numbers involved are all multiples of five. The following example shows how to filter a database to identify only those molecules that pass all four of the Lipinski criteria. The values of the Lipinski descriptors are also added to the output file as data fields. Note that whereas molecular weight is directly available as an attribute of a Molecule, and LogP is available as one of the three group contribution descriptors calculated by OpenBabel, we need to use SMARTS pattern matching to identify the number of hydrogen bond donors and acceptors. The SMARTS patterns used here correspond to the definitions of hydrogen bond donor and acceptor used by Lipinski:

import pybel

HBD = pybel.Smarts("[#7,#8;!H0]")

HBA = pybel.Smarts("[#7,#8]")

def lipinski(mol):

   """Return the values of the Lipinski descriptors."""

   desc = {'molwt': mol.molwt,

      'HBD': len(HBD.findall(mol)),

      'HBA': len(HBA.findall(mol)),

      'LogP': mol.calcdesc(['LogP']) ['LogP']}

   return desc

passes_all_rules = lambda desc: (desc ['molwt'] <= 500 and

         desc ['HBD'] <= 5 and desc ['HBA'] <= 10 and

         desc ['LogP'] <= 5)

if __name__=="__main__":

   output = pybel.Outputfile("sdf", "passLipinski.sdf")

   for mol in pybel.readfile("sdf", "inputfile.sdf"):

      descriptors = lipinski(mol)

      if passes_all_rules(descriptors):

         mol.data.update(descriptors)

         output.write(mol)

   output.close()

### Future work

The future development of Pybel is closely linked to any changes and improvements to OpenBabel. With each new release of the OpenBabel API, the SWIG bindings will be updated to include any additional functionality. However, additions to the Pybel API will only occur if they simplify access to new features of the OpenBabel toolkit of general use to cheminformaticians. In general, the Pybel API can be considered stable, and an effort will be made to ensure that future changes will be backwards compatible.

## Conclusion

Pybel provides a high-level Python interface to the widely-used OpenBabel C++ toolkit. This combination of a high performance cheminformatics toolkit and an expressive scripting language makes it easy for cheminformaticians to rapidly and efficiently write scripts to manipulate molecular data.

Pybel is freely available from the OpenBabel web site^2 ^both as part of the OpenBabel source distribution and for Windows as an executable installer. Compiled versions are also available as packages in some Linux distributions (openbabel-python in Fedora, for example).

## Availability and Requirements

**Project name: **Pybel

**Project home page: **

**Operating system(s): **Platform independent

**Programming language: **Python

**Other requirements: **OpenBabel

**License: **GNU GPL

**Any restrictions to use by non-academics: **None

## Authors' contributions

GRH is the lead developer of OpenBabel and created the SWIG bindings. NMOB developed Pybel, and extended the SWIG interface file. CM compiled the SWIG bindings on Windows and added convenience functions to the OpenBabel API to facilitate access from scripting languages. All authors read and approved the final manuscript.

## Supplementary Material

Additional file 1 Pybel API. The HTML documentation of the Pybel API (application programming interface). Click here for file
